# Supplementary material for: A Systematic Review of the Relationship between Chest CT Severity Score and Laboratory Findings and Clinical Parameters in COVID-19 Pneumonia
Source: Diagnostics (Basel). 2023 Jun 29;13(13):2223. doi: 10.3390/diagnostics13132223 (PMC10340676; doi:10.3390/diagnostics13132223)
Supplement: Supplementary file 1 [file diagnostics-13-02223-s001.zip › diagnostics-2445439-supplementary.pdf]

Table S1. Characteristics of included studies showing the correlation between CT severity score and both laboratory findings and clinical parameters.

Authors	Location/Country	Participants	Age	Imaging parameters	Main findings	CT severity score calculation
(1) Zinellu et al., 2021	The University Hospital of Sassari, Italy	50 COVID-19 patients (18F/32M)	66.9+14.7	The paper does not provide detailed information on the imaging parameters used in this study. However, it mentions that routine imaging parameters were collected at admission along with other clinical and laboratory data to identify factors that predict length of hospital stay in COVID-19 patients. The specific type of imaging performed is not specified but given the focus on lung damage and hypoxia as variables, it is likely that chest X-rays or CT scans were included among the routine imaging tests performed.	The results showed that COVID-19 patients with prolonged hospitalization (PHS) had significantly lower PaO2/FiO2 ratio and lymphocytes, and significantly higher Chest CT severity score, C-reactive protein (CRP), and lactic dehydrogenase (LDH) when compared to non-PHS patients. In univariate logistic regression analysis, Chest CT severity score, intensity of care required during hospitalization, PaO2/FiO2 ratio on admission, CRP levels at admission, and platelet to lymphocyte ratio were all found to be significant predictors of PHS. However, in multivariate logistic regression analysis only the Pa02/Fi02 remained a significant predictor for PHS with an odds ration 0f 0.9164; 95% CI: 0.8479-0.9904; p= .0275). The researchers also identified a threshold value for predicting prolonged hospital stay using ROC curve analysis which was less than or equal to248 mm Hg for the Pa02/ Fi02ratio on admission	The paper does not provide a detailed description of the CT severity score calculation used in this study. However, it mentions that Chest CT severity score was one of the variables collected at admission and found to be significantly associated with prolonged hospital stay in COVID-19 patients.
(2) Padmanaban et al., 2022	India	100 COVID-19 patients (75 M, 25 F)	52+13	The paper does not provide a detailed description of the imaging parameters used in this study. However, it mentions that all patients underwent chest CT scans	The main results are: 1. Patients with higher CT severity scores had more severe COVID-19 disease, as indicated by longer hospital stays, higher rates of ICU admission, and increased mortality. 2. Blood sugar levels at presentation were significantly associated with both disease severity (as measured by CT severity score) and	The CT severity score was calculated based on the extent of lung involvement observed in chest CT scans using the COVID-19 Reporting

				using a 64-slice multi-detector CT scanner. The images were evaluated by two radiologists who were blinded to clinical information and scored based on the extent of lung involvement using the COVID-19 Reporting and Data System (CO-RADS) scoring system. The CO-RADS score ranges from 1 to 5, with higher scores indicating more severe disease involvement. Beyond this information, no further details about specific imaging parameters such as slice thickness or radiation dose are provided in this paper.	clinical outcomes such as length of hospital stay and need for mechanical ventilation. 3. Adequate glycemic control is essential in preventing further COVID-19 complications. Overall, the study suggests that both CT severity score and blood sugar level at presentation can serve as useful prognostic markers for predicting COVID-19 patient outcomes.	and Data System (CO-RADS) scoring system. The CO-RADS score ranges from 1 to 5, with higher scores indicating more severe disease involvement. In this study, two radiologists who were blinded to clinical information evaluated each patient's chest CT scan images and assigned a CO-RADS score based on their observations of lung involvement. If there was any disagreement between the two radiologists' scores, a third senior radiologist reviewed the images and provided a final consensus score for that patient. The mean value of these scores was used as an overall measure of disease severity for each patient in

						subsequent analyses.
(3)<br>Fois et al., 2020	The University Hospital of Sassari, Italy	119 COVID-19 patients (42 F, 78M)	71 (58-80 years)	There are no CT imaging parameters mentioned in this study. The study investigated different inflammation indexes derived from complete blood cell count (CBC) to predict in-hospital mortality of COVID-19 patients.	The main results are: 1. The Systemic Inflammation Index (SII) on admission can independently predict in-hospital mortality in COVID-19 patients and may assist with early risk stratification for this group. 2. non-survivors had significantly higher values of several inflammation indexes, including AISI, dNLR, NLPR, NLR, SII and SIRI when compared with survivors. 3. After adjusting for confounders only the SII remained significantly associated with survival according to multivariate Cox regression analysis. Overall, these findings suggest that measuring systemic inflammation using CBC-derived indexes such as the SII could be a useful tool for predicting outcomes in COVID-19 patients and identifying those at highest risk of mortality who may require more intensive management or monitoring	There is no information on how CT severity score was calculated in this study. The study investigated different inflammation indexes derived from complete blood cell count (CBC) to predict in-hospital mortality of COVID-19 patients
(4)<br>Younus et al., 2022	Nishtar Hospital, Multan, Pakistan	original 4180 (4004 in this study, 2662M, and 1342F)	The age range of patients included in this study was 18-90 years old. The median age of patients was 47 years old, and there were slightly	Chest CT imaging was performed on a 64-detector CT scanner (Toshiba). All patients were examined in supine position. CT scan had following parameters: X-ray tube parameters - 120 KVp, 350 mAs; pitch - 1.0; rotation time - 0.5sec; section thickness – 5 mm; intersection space – 5 mm.	The main results are that the novel CT severity score (CTSS) had a high sensitivity and specificity for diagnosing COVID-19 pneumonia. - Patients with higher CTSS scores tended to have more severe clinical stages of the disease, longer hospital stays, and a higher risk of mortality compared to those with lower scores. - Raised CRP levels (>0.5 mg/dL) were found in 81.86% and increased D-dimer levels (>500 ng/mL) were found in 92.3% of patients. - The most common radiological findings on chest CT scans included ground-glass opacities which were observed in almost all cases (98%). -The authors concluded that this novel CT scoring system might aid risk stratification and short-	The CT severity score (CTSS) was calculated based on the extent and severity of lung involvement observed on chest CT scans. The scoring system used in this study assigned points to each lobe of the lungs based on the percentage of lung involvement, as follows: - 0 points: no involvement - 1 point:

			more male than female patients (52% vs. 48%).		term prognostication for patients suffering from COVID-19 pneumonia	<5% involvement - 2 points: 5%-25% involvement - 3 points: >25%-50% involvement - 4 points:>50% The scores for all five lobes were then added together to obtain a total CTSS ranging from zero to twenty-five. A higher score indicated more severe disease with greater lung damage. In this study, two radiologists independently reviewed all chest CT scans and assigned scores using this semi quantitative scoring system. Any discrepancies between their readings were resolved by consensus or adjudication by a third reader if necessary
(5) Vishwanath et al., 2022	The study was conducted in Pune, Maharashtra,	178 (98 F, 80 M)	30-62 ears	All patients underwent chest HRCT examination at Sri Jayadeva Institute	The main result of the study was that there was a significant association between chest CT severity scores (CTSS) and vaccination status, age, gender, co-morbidities, and stage of disease	The CT Severity Score (CTSS) was calculated based on the COVID-19 Reporting and

	India.			of Cardiovascular Sciences using Philips 128 slice CT scanner. Patients were placed in a supine position and with single breath hold scanning was performed. Sagittal and coronal reformatted images were subsequently obtained. Scanning parameters were as follows scan direction (craniocaudal), tube voltage (120kV), tube current (251mA), slice collimation (64×0.625), no contrast material was used. The results for the chest HRCT images were collected and evaluated using the Picture Archiving and Communication Systems (PACS)	in the cohort of COVID-19 patients. The study found that fully vaccinated patients had lower mean CTSS compared to unvaccinated or partially vaccinated patients. Therefore, full vaccination can aid in reducing the severity of lung involvement in COVID-19 infection.	Data System (CO-RADS) categories assigned to each patient's chest CT scan. The CO-RADS categories range from 1 to 5, with higher scores indicating greater likelihood of COVID-19 infection. For patients who were classified as CO-RADS category 4 or 5, a semi-quantitative assessment of pulmonary involvement was performed by dividing each lung into three zones and assigning a score ranging from zero to five for each zone based on the percentage of lung involvement: - Zero = no involvement - One = <25% involved - Two = between 26% and 50% involved - Three = between >50% and <75% - Four= ≥75% The total CTSS for each patient was then calculated

						by summing up the individual scores for all six zones in both lungs, resulting in a possible maximum score of thirty points per patient.
(6) Lin et al., 2021	China	56 COVID-19 patients	46.54 ±15.82	Following admission, all patients underwent high-resolution plain chest CT scanning utilizing a Siemens 64-row CT scanner located in Erlangen, Germany. The study employed specific scanning parameters, including a voltage of 120 kV, a current range of 100-280 mA, a layer thickness and interlayer spacing of 5 mm, a collimation of 5 mm, a screw pitch of 1 mm, a field of view measuring 30 cm, and a matrix of 512 × 512. The lung window width was set at +1500 Hu with a corresponding lung window level of −500 Hu, while the mediastinal window width was +300 Hu with a mediastinal window level of +10 Hu. Multiplanar reconstruction was performed on certain patients based on the	The main findings of the study are that there is a correlation between chest CT severity score and laboratory indicators (C-reactive protein, erythrocyte sedimentation rate, and LDH) in patients with COVID-19. The study suggests that these indicators may be useful for assessing disease severity in patients with COVID-19. Additionally, the CT score was found to be higher in severe cases compared to moderate or mild cases.	The CT severity score was calculated based on the extent of lung involvement and presence of certain features associated with COVID-19 pneumonia. The scoring system used in this study ranged from 0-25 and was based on several factors including ground-glass opacities, consolidation, crazy-paving pattern, air bronchogram sign, pleural effusion or thickening and lymphadenopathy. Two radiologists who were blinded to clinical information scored each chest CT image independently using this system. The scores assigned

				results of their chest CT scans. The reconstruction parameters utilized were as follows: a layer thickness of 1.0 mm, an interlayer spacing of 0.7 mm, a lung window of B70f, and a reconstruction function of D30f.		by the two radiologists were averaged to obtain a final score for each patient's chest CT images.
(7) Smet et al., 2020	AZ Delta General Hospital, a tertiary network hospital in Roeselare, Belgium	186 COVID-19 patients (77F, 109M)	69 (52-80 years)	All patients were imaged by MDCT using either of the following CT scanners: the GE LightSpeed VCT scanner (1-mm slice thickness), Siemens Somatom AS (1-mm slice thickness) or the GE Optmima 660 scanner (1.25-mm slice thickness). All scans were performed without intravenous contrast with the patient in the supine position during end-inspiration. Only the initial CTs were included; follow-up CTs during the study time window were not analyzed.<br>Image Viewing and Evaluation Two cardiothoracic radiologists with 24 and 9 years of	The main findings of the study are: 1. 59% of COVID-19 patients were vitamin D deficient on admission. 2. Male patients with advanced stages of COVID-19 pneumonia had progressively lower levels of serum 25(OH)D. 3. Vitamin D deficiency on admission was associated with mortality in COVID-19 patients, independent of age, chronic lung disease, and extent of lung damage expressed by chest CT severity score. Overall, the study suggests that low levels or deficiency in serum 25(OH)D may be a risk factor for severe outcomes in COVID-19 patients and highlights the importance to monitor vitamin D status as part their clinical management plan during hospitalization period.	Percentage of pulmonary tissue affected by COVID-19 pneumonia was expressed as CT severity score on a scale from 0 to 25, calculated by scoring loss of well-aerated functional lung tissue for each individual pulmonary lobe on a scale from 0 to 5 and summating this score for the five lobes.

				experience retrospectively reviewed the CT exams on a PACS workstation (IDS7, Sectra) with multiplanar reconstruction tools. Decision was reached by consensus.		
(8) Yazdi et al., 2021	The study is being conducted at the Imam Khomeini Hospital, Tehran University of Medical Sciences, Tehran, Iran.	478 participants	53.92 (15.4).	The CT scan protocols or parameters used were 120 kVp tube voltage; 50–150 mAs tube current; 0.75 s tube rotation time; 0.5–0.75 s gantry rotation time; 2–3-mm section thickness; and 0.6–2 mm beam collimation.	The results showed that among 478 participants in the study, 73.6% were admitted to the hospital and 8.7% were admitted to the ICU. Myalgia, fever, and dyspnea were the most common symptoms. A review of chest CT scans showed that Ground Glass Opacity and consolidation were the most patterns of lung lesions. Initial assessment of COVID-19 patients, including symptoms, vital signs, and routine laboratory tests, can predict the severity of lung involvement and unfavorable outcomes. The total chest CT score was significantly higher in patients with anosmia, higher respiratory rate, higher C-reactive protein, higher white blood cell count, and lower SpO2. It was also associated with a higher risk of ICU admission, longer hospital stays, and longer recovery time.	The CT severity score was calculated by summing the scores of each of the five lobes of lungs, which were assigned a score from 0 to 5 based on the extent of involvement (0, no involvement; 1, < 5% involvement; 2, 5–25% involvement; 3, 26–49% involvement; 4, 50–75% involvement; 5, > 75% involvement). The total chest CT score ranged from 0 to 25.
(9) Arcari et al., 2022	the location of conducting this study This was a single-center observational study conducted at the authors' institution	112 patients (69 males (62%), and 43 females (38%)	The mean age of the study population was 67 with a range of 16 years.	This study used two multidetector CT scanners (Philips Brilliance 16 and Brilliance 64) for all examinations. Scanning parameters were set as indicated by the manufacturer's standard recommended presetting	The main result of the study was that a semiquantitative chest computed tomography (CT) score was found to be an independent predictor of noninvasive positive-pressure ventilation (NPPV) failure and in-hospital mortality in patients hospitalized for COVID-19 pneumonia. The CT score accurately identified patients who responded poorly to NPPV. There was no statistically significant difference	The CT severity score was calculated by using a semiquantitative severity score described in the literature, considering the degree of anatomic

	(Vannini hospital), India			for a thorax routine. The authors acquired images with a 1-mm slice thickness and a reconstruction increment of 0.5 mm in all patients using a soft tissue kernel of B20 and a lung kernel of B60. Coronal and sagittal multiplanar reconstructions (MPR) were performed in all cases.	between males and females in any of the groups (venturi mask, NPPV responders, and NPPV failures).	involvement (0: no involvement; 1: < 5% involvement; 2: 5-25% involvement; 3: 26-50% involvement; 4: 51-75% involvement; and 5: > 75% involvement). The involvement of each lobe was assessed by the identification of abnormalities (ground-glass opacity, crazy-paving, and consolidation) and then visual estimation of their extent (eyeballing). The resulting global CT score was the sum of each individual lobar score (0-25).
(10) Inamdar and Saboo, 2022	India	30 patients with confirmed COVID-19	The mean age of the participants was 46.7 (20.1) years.	The article stated that chest CT scans were obtained for all participants, but did not specify the type of CT scanner used, the imaging parameters (e.g. tube voltage, tube current, slice thickness, etc.), or the specific imaging protocol (e.g. contrast-enhanced or non-contrast-enhanced). The article also did not	there is a positive correlation between chest CT severity score and blood glucose levels in patients with COVID-19. The study found that patients with higher chest CT severity scores tended to have higher blood glucose levels, and patients with diabetes or hyperglycemia tended to have higher chest CT severity scores. These findings suggest that glucose metabolism may play a role in the severity of COVID-19 pneumonia. However, the study has limitations, including a small sample size and lack of detailed information on the imaging protocols	the article stated that the CT severity score was calculated using a semiquantitative scoring system based on the extent of lung involvement observed in the chest CT scan. According to the article, the score ranged from 0 to 25, with higher scores

				provide information on the interpretation of the chest CT scans or the criteria used for the semiquantitative scoring of lung involvement. The lack of detailed information on the imaging parameters or protocols used in the study may limit the ability to replicate or compare the results of the study.	and data collection methods used. These findings should be interpreted with caution and further research is needed to confirm and expand upon these findings.	indicating more severe lung involvement.
(11) Zhou et al., 2020	China	134 COVID-19 patients	The median age was 48 years (range, 14–84 years) and 58 years (range, 33–95 years) for survivors and non-survivors, respectively.	Tube voltage 120 kV, automatic tube current modulation (40 mA), pitch 0.99–1.22 mm, matrix 512×512, slice thickness 5–10 mm, field of view, 350 mm × 350 mm, reconstruction slice thickness 0.625 or 1.25 mm.	The results demonstrated that the total CT severity scores were significantly greater in the deceased group than those in the recovered group for each of the five lobes and the whole lung. The mortality risk was increased by 6.9 times in patients with total CT scores of 16 points or more compared to those with less than 16 points. The optimal cutoff value of the total CT scores was 16.5 points, achieving 69.4% sensitivity and 82.2% specificity for the prognostic prediction.	The CT severity score was calculated by summing up the highest severity scores and severity scores from different disease stages of lung involvement based on chest CT imaging.
(12) Valk et al., 2022	This study was conducted in the intensive care units (ICUs) of two tertiary centers, the Academic	254 COVID-19 patients	The median age was 65 [60–72] years.	The imaging parameters or protocols included APACHE II score, FiO2, PEEP, Pmax, RR, VT, and the blood gas analysis results.	The CTSS had an association with hospital mortality, 28-day, and 90-day mortality, but not with length of stay or duration of ventilation. The RALE score was not associated with any of the secondary outcomes. The main findings of this study were that the CT-based score was significantly higher in non-survivors and	The CT severity score was calculated by trained scorers using the Radiographic Assessment of Lung Edema (RALE) score and the chest CT

	Medical Center, and the Free University Medical Center, Amsterdam, The Netherlands.				showed an association with all mortality outcomes. The CTSS had an association with hospital mortality, 28-day, and 90-day mortality, but not with length of stay or duration of ventilation. The RALE score was not associated with any of the secondary outcomes.	Severity Score (CTSS) of the first available CT scan. The CTSS was calculated using a system for scoring ground-glass opacity, interstitial opacity, and air trapping on thin-section CT scan.
(13) Abbasi et al., 2021	The study was performed in a large tertiary referral academic hospital designated to COVID-19 patients located in Mashhad, Iran.	This study included 262 adult patients (aged 18 and older) hospitalized with laboratory-confirmed COVID-19 infection	The median age of all patients was 58 (with an interquartil e range of 43-67). The median age of survivors was 56 (with an interquartil e range of 41-65) and the median age of deceased patients was 66 (with an interquartil e range of 58-77).	Patients were examined in the supine position with both arms extended above the head. The scans were taken in the caudocranial direction, spanning the entire chest from the diaphragmatic dome up to the lung apices. All scans were performed without intravenous contrast administration. All chest CT images were transferred from PACS into a dedicated workstation and analyzed by OSIRIX MD ™ (version 10.0.1) software and a medical monitor. From each dataset, multiplanar reconstructions were generated on the standard three orthogonal planes — axial, coronal, and sagittal.	The results showed that mortality was significantly higher in patients with higher CT severity score even after adjustment for clinical, demographics and laboratory parameters. The main findings were that in univariable analysis, odds of in-hospital death were higher in patients with diabetes, hypertension, renal failure or coronary heart disease, age, hypoxemia at admission, lymphocyte percentage less than 20%, and elevated LDH. Additionally, higher CT severity score and crazy paving pattern on the CT at admission were associated with higher odds of in-hospital death. Multivariate regression analysis showed increasing odds of in-hospital death associated with older age and the presence of coronary artery disease.	The CT severity score index calculation is based on a semi-quantitative scoring method previously used by Zhou et al. [8] and Wang et al. [4] to score the degree of involvement using a system previously described for severity of acute respiratory distress syndrome on thin section lung CT. The score is based on the number of lung zones affected and the degree of involvement in each zone.

(14) Halmaciu et al., 2022	The location of this study is Targu-Mures, Romania.	267 COVID-19 patients	71.19 ± 10.25 years, with a range of 33-94 years. The mean age of survivors was 70.01 ± 8.99 years, while the mean age of non-survivors was 73.85 ± 12.29 years	The study mentions that chest CT exams were performed within the first 24 hours of admission. The images were analyzed using a PACS (Picture Archiving and Communication System) workstation. The CT images were evaluated to assess the extent of pulmonary parenchymal involvement for the presence of ground-glass opacities (GGOs), consolidation, and pleural effusion. The Total Severity Score (TSS) was calculated by quantifying the disease-affected areas for each lobe to evaluate pulmonary parenchymal involvement. Each of the five lobes was given a score ranging from 0 to 4, based on the percentage of the affected area as none (0%), minimal (1–25%), mild (26–50%), moderate (51–75%), or severe (76–100%). TSS was calculated by adding the values for five lobes ranging from 0 to 20. The study does not provide further details on the CT	The main findings of this study are that a high baseline value for inflammatory biomarkers (MLR, NLR, SII, SIRI, AISI, and IL-6) and TSS (Chest CT Severity Score) is a strong predictor of adverse outcomes, including the need for invasive mechanical ventilation (IMV) and mortality, in patients with COVID-19 pneumonia. The study also found that patients above 70 with AF, dyslipidemia, and unvaccinated status highly predicted IMV need and fatality. Likewise, CHF and CKD were independent predictors of increased mortality. The study suggests that the use of hematological markers and the lung damage score in medical practice allows for better stratification of risk groups and the establishment of appropriate therapeutic management, thus improving the progression of patients with COVID-19.	The Chest CT Severity Score (TSS) was calculated by quantifying the disease-affected areas for each lobe to evaluate pulmonary parenchymal involvement. Each of the five lobes was given a score ranging from 0 to 4, based on the percentage of the affected area as none (0%), minimal (1–25%), mild (26–50%), moderate (51–75%), or severe (76–100%). TSS was calculated by adding the values for five lobes ranging from 0 to 20. The higher the TSS, the greater the extent of pulmonary involvement. The TSS is a widely used method for quantifying the severity of lung involvement in COVID-19 patients and has been shown to be a useful predictor of disease

				imaging parameters or protocols used.		severity and mortality.
(15) Abd El Megid et al., 2022	The study was conducted in the Radiology Department and Chest Department of the Faculty of Medicine at Zagazig University in Zagazig City, Sharkia Governorate, Egypt. This information is mentioned in the author details section of the document.	the study included 305 patients with confirmed COVID-19 infection	what is the age or age range The mean age of the patients in this study was 41.9 years, with a range of 18-73 years.	the chest CT scans were performed using a 128-slice CT scanner with the following imaging parameters: slice thickness of 0.625 mm, slice interval of 0.625 mm, and images were acquired in supine position and deep inspiration. The CT scan images were then reconstructed. Two competent radiologists assessed all patients' chest CT scans at the same time and calculated the chest CT severity score for patients.	The main findings of this document are that chest CT scans can help clinicians in developing a management strategy and serve as a predictor of illness severity and possible outcomes in individuals with COVID-19 infection. The severity of a chest CT scan is positively correlated to inflammatory markers and oxygen demand. The study also found that there was a significant reverse relationship between CT severity score and blood oxygen saturation level which has major clinical implications. The study suggests that in patients with COVID-19 infection, clinicians should pay closer attention to chest CT severity scores. Additionally, the study found that risk factors such as hypertension, diabetes, lung illness, and coronary artery disease are associated with a bad prognosis, with an even poorer outcome when several risk factors are present.	the chest CT severity score was assigned for each lobe as follows: 0 for no involvement, 1 for < 5% involvement, 2 for 5–25% involvement, 3 for 25–50% involvement, 4 for 50–75% involvement, and 5 for > 75% involvement. The overall severity score was calculated by summation of scores from all five lung lobes. A mild grade was defined as 0–7 points, a moderate grade was defined as 8–16 points, and a severe grade was defined as 17–25 points. The sum of points in each lobe was used to calculate the final score, which was used to calculate the CT severity score. Two competent radiologists assessed all patients' chest CT scans at the same

						time and calculated the chest CT severity score for patients.
(16)<br>Atre et al., 2022	India	1002 patients enrolled in a study on COVID-19	The mean age of the patients was 49.0 years, with a range of 18-90 years.	non-contrast chest HRCT scans of the COVID-19 patients were performed on a multidetector CT scanner (Philips Ingenuity 128 Slice CT; Philips Healthcare, Amsterdam, Netherlands and GE 32 Slice; GE Healthcare, Waukesha, USA) with the patient in supine position, during end inspiration. Scanning parameters were in line with the manufacturer's standard recommendations for a routine thorax scan. All CT images were reconstructed to thin slices using the Multiplanar Reformatting (MPR) technique. Appropriate infection prevention and control measures were arranged for the CT technologists and the patients. The HRCT images of the COVID-19 patients were independently examined on standardized workstations, by two radiologists with 15 years'	The main findings of the document are:<br><br>1. The severity of lung involvement in COVID-19 patients, as measured by the CT severity score (CTSS), was significantly associated with the vaccination status, age, gender, co-morbidities, and stage of disease.<br><br>2. Patients in the early stages of the disease had significantly lower mean CTSS and milder grades of CT scores compared to patients in later stages of the disease.<br><br>3. The majority (76.6%) of the study patients were unvaccinated, and breakthrough infections occurred in 4.4% of the patients.<br><br>4. Patients with co-morbidities had a higher mean CTSS and a greater percentage of cases with moderate and severe grades of CTSS compared to patients with no co-morbidities.<br><br>5. The study found a higher mean CTSS and a greater percentage of cases with moderate and severe grades of CTSS in the middle-aged (45-59 years) and elderly (>60 years) population.<br><br>6. Men had a higher mean CTSS and a greater percentage of cases with moderate and severe grades of CTSS compared to women.<br><br>7. The study recommends further multicentric	The CT severity score (CTSS) was calculated based on the extent of anatomic involvement of each of the five lobes of the lungs, using a semi-quantitative scoring system. The scoring system ranged from 0 to 5, with 0 indicating no involvement and 5 indicating involvement of more than 75% of the lobe. The individual lobar scores were then summed up to obtain the total CTSS, which ranged from 0 (no involvement) to 25 (maximum involvement). Based on the total CTSS, the severity of lung involvement in the patients was further graded into mild (CTSS of 0-8), moderate (CTSS of 9-15), and severe (CTSS of 16-25) categories.

				experience in reporting chest CT images.	studies involving larger groups of COVID-19 patients to validate the results of this study and to evaluate the impact of SARS-CoV-2 vaccination on the overall patient outcomes.	The CTSS was used to evaluate the severity of lung involvement in COVID-19 patients and to assess the association of CTSS with age groups, gender, co-morbidities, and vaccination status.
(17) Aziz-Alhari et al., 2022	This study was conducted at Rasoul Akram Hospital, Tehran, Iran.	148 patients	The mean age of the patients in this study was 59.27 ± 16.80 years (range 20–98 years).	The imaging parameters and protocol for the CT scan in this document are as follows: a low-dose lung CT without contrast was performed using a 16-slice CT-scanner (Toshiba Aquilion; Toshiba Medical System, Japan). The image acquisition parameters were a tube voltage of 120 KVp, a tube current of 60 mAs, and a slice thickness of 5 mm at a 5-mm slice interval. The Adaptive Iterative Dose Reduction Algorithm was used to reconstruct the images. The mean CT dose index volume was 3.8 mGy (range 2.5–6.5 mGy). All CT images were interpreted in both pulmonary (width: 1600 HU, level: –550 HU) and	The main findings of this document are:<br><br>1. Ground glass opacities (GGO) were the most common CT finding in COVID-19 patients (95.9%), followed by consolidation (70.9%) and bilateral lung involvement (95.9%).<br>2. The predominant pattern of distribution was diffuse peripheral/central involvement (71.6%).<br>3. Pleural effusion, pulmonary nodules, lymphadenopathy, atelectasis, fibrosis, and pericardial effusion were rare.<br>4. Right lung involvement was slightly predominant over left lung involvement.<br>5. The CT severity score was higher in severe/critical patients, and there was a significant difference between the presence of pleural effusion and the severity of disease.<br>6. The mortality rate was significantly higher in patients with comorbidities, including diabetes mellitus and hypertension.<br>7. Laboratory parameters such as lymphocyte counts, WBC, neutrophil counts, lactate dehydrogenase, urea, CRP, PT, troponin, and D-dimer were significantly associated with disease severity and mortality.	The CT severity score in this study was calculated using a semi-quantitative scoring method. The severity of CT findings was assessed in all lobes of the lung, and a score was assigned based on the extent of involvement. The score ranged from 0 to 5 for each lobe, with a maximum possible score of 25 for all five lobes. The severity score was then categorized into three groups: moderate (score 0-7), severe (score 8-14), and critical (score 15-25). The optimal CT severity score

				mediastinal (width: 300 HU, level: 45 HU) windows by 2 different expert radiologists separately. In cases with conflict in interpretations, the opinion of the senior attending radiologist applied.	8. The most common symptoms were cough, respiratory distress, dyspnea, and fever. 9. Hydroxychloroquine, levofloxacin, and lopinavir/ritonavir were the most frequent medications used. 10. The study involved 148 patients with positive SARS-CoV-2 RT-PCR tests, and the mortality rate was 37%.	threshold for predicting mortality was found to be 15.5 with a sensitivity of 61.8% and a specificity of 76.3%. The best CT severity score cut-off for predicting the severity of disease was 12.5 with a sensitivity of 68.3% and a specificity of 72.7%.
(18) Saeed et al., 2021	The study was conducted at Shifa International Hospitals in Islamabad, Pakistan.	103 consecutive patients who were RT-PCR positive for COVID-19.	The mean age of the participants was 30.55 ± 7.73 years. The study involved 81 male and 22 female patients	The imaging parameters or protocols used in the study are mentioned in subsection [1] of the document. The high-resolution CT scans were performed on a 320 slice CT scanner dedicated for known or suspected COVID patients. The imaging parameters were tube current of 20mAs, voltage 100kVp, slice thickness of 1mm, and scan time of 3.32 seconds. All patients were supine and CT acquisition made in full inspiration with recorded verbal commands from costophrenic angle to the apex of the lung. Non-	The main findings of the study are: <br>1. The CT severity score (CT-SS) can be used as a semi-quantitative way of assessing the severity of COVID-19 in the initial high-resolution chest CT. The CT-SS score of 19.5 in this study may suggest that the severe and critical forms of the disease, with a fair degree of confidence, can be detected. <br>2. The severity of COVID-19 pneumonia-related overall hospitalization, mortality, and morbidity are relatively less severe in Pakistan. <br>3. A linear relationship between CT severity score and symptomatology of the patients was noticed. These symptoms defined ICU admission and a hospital stay of these patients. <br>4. The study showed that patients in the severe group had reduced lymphocytes and increased	The CT severity score (CT-SS) is calculated by adding the scores of all 20 individual regions of both lungs combined, with a range from 0 to 40 points. The scoring of each region is done subjectively by the radiologists, who attribute a score of 0, 1, or 2 to each region, depending on the degree of parenchymal opacification. A score of 0 is given if there is no opacification, a score of 1 is given if there is less than 50%

				contrast technique was used in the acquisition of CT images. Kernel BI57 for lungs and Kernel Bf37 for mediastinum were used with SAFIRE strength 2 for both. Two consultant radiologists, having experience of 7 to 10 years in body imaging, evaluated the HRCT studies in consensus.	neutrophils and raised CRP levels.<br><br>5. The study involved 81 male and 22 female patients and showed that 74 cases were above 50 years of age with only 29 below 50 years.<br><br>6. The study found a significant difference in the scoring of lung opacity in mild and severe groups in each lung segment, with significant variables being right lower lobe's medial and lateral basal segment, left upper lobe's superior lingula segment, and left lower lobe's medial basal and lateral basal segments.	opacification, and a score of 2 is given if there is 50% or more opacification. The CT-SS score of 19.5 in this study suggests that severe and critical forms of the disease can be detected with a fair degree of confidence.
(19)<br>Padelli et al., 2021	The study was conducted at the Martinique University Hospital, which is in Martinique, a French overseas territory in the Caribbean Sea.	The study included a total of 64 Afro-Caribbean patients hospitalized at the Martinique University Hospital between February 1, 2020, and February 28, 2021, with RT-PCR-confirmed COVID-19 and complete medical and	The mean age of patients in the study was 68 years	This study does not provide detailed information on the CT scan parameters or protocols. The study only mentions that a radiological severity score of 0-4 was assigned to each lung depending on the extent of involvement by consolidation or ground glass opacities. The score was calculated by summing the five lobe scores (range, 0-20), and a simplified severity score ranging from mild (< 7), moderate (8-17), and severe (18 or more) was used. This suggests that chest CT scans were performed on the patients to assess the extent of lung	The document contains various results related to the study on the prediction of COVID-19 deterioration using suPAR levels, chest CT severity score, and other biomarkers. Some of the key findings include:<br><br>- Plasma suPAR levels at admission have potent predictive value for COVID-19 outcome, with an ROC-derived cutoff of 8.95 ng/ml.<br>- Patients with higher radiological severity scores on CT scan had a higher risk of clinical deterioration and worse outcomes.<br>- Chest CT scan severity score, NEWS2, and suPAR were found to be independent risk factors for the incidence of events (i.e., ICU admission or death) during follow-up.<br>- The best values of NEWS2 and plasma suPAR concentration to predict outcome were, respectively, a NEWS2 score of 3 and suPAR plasma level of 8.95 ng/ml.<br>- The study found that clinical and laboratory parameters previously reported to have	In this study, a radiological severity score of 0-4 was assigned to each lung depending on the extent of involvement by consolidation or ground glass opacities. The score was calculated by summing the five lobe scores (range, 0-20), and a simplified severity score ranging from mild (< 7), moderate (8-17), and severe (18 or more) was used. The CT severity score was used to assess the extent of lung involvement by

		biological records.		involvement by COVID-19. However, the document does not provide information on the specific CT scan parameters or protocols used for the chest CT scans.	prognostic significance in COVID-19 patients may be used to predict clinical deterioration in Afro-Caribbean people.	COVID-19 and to predict the risk of clinical deterioration and worse outcomes. Patients with higher CT severity scores had a higher risk of clinical deterioration and worse outcomes. The CT severity score was found to be an independent risk factor for the incidence of events (i.e., ICU admission or death) during follow-up in COVID-19 patients.
(20) Sharma et al., 2022	The study is done in St. Stephens Hospital, Tis Hazari, Delhi, India.	150 patients with COVID-19 infection	The mean age of the study group was 54.46 (16.71) years. Most cases were aged between 41 and 60 years, followed by 61–80 years.	The imaging parameters were a Philips incisive 128 MDCT scanner with a slice thickness of 0.625–1.250 mm and patients in the supine position. The CT scan parameters or protocols used in this study are tube voltage of 120kvp, automatic tube current modulation (tube current) of 140–200mAs, pitch of 0.99–1.22 mm matrix, 512 *512 slice thickness of 5 mm, and field of view of 350*350 mm.	The results of the study showed that the mean CRP was significantly higher in the severe group (11.7) compared to the mild (5.3) and moderate (5.2) groups, and this finding had a positive statistically significant correlation with CTSS (p 0.045). The mean leukocyte count increased with increasing disease severity, but this correlation was not statistically significant (p 0.173). The laboratory tests showed raised CRP (CRP > 0.1 mg/dL) in 78.7% of patients, raised ESR (ESR > 10 mm per hour) in 68.7%, lymphopenia (< 1–3 × 10 3 /μL) in 52% cases, increased D-dimer (> 0–200 ng/mL) in 62% patients, leucocytosis (WBC count > 4–10 × 10 3 /μL) in 48.7% patients, and increased serum ferritin (> 23.9–336 ng/mL) in 28.7% patients. Mortality rate of COVID-19 patients in this study was associated with higher	The CT severity score calculation in this study is based on the sum of the individual lobar scores, which range from 0 (no involvement) to 25 (maximum involvement), when all the five lobes show more than 75% involvement.

					CT severity grade, with the mean CT severity score among patients who died being significantly higher than patients who survived. There is a significant statistical correlation between CT severity grade and patient survival, and CT severity score is positively correlated with lymphopenia and raised CRP, D-dimer, and serum ferritin levels.	

References

1. Zinellu A, De Vito A, Scano V, Paliogiannis P, Fiore V, Madeddu G, et al. The PaO2/FiO2 ratio on admission is independently associated with prolonged hospitalization in COVID-19 patients. J Infect Dev Ctries. 2021;15(3):353–9.

2. Padmanaban U.B., Kumar G.S., Chundeli M.I., Afroze M.K.H. Clinical Spectrum Of COVID19 Patients Admitting To A Tertiary Care Hospital And Their Outcome Based On Ct Severity Score And Blood Sugar. Res J Pharm, Biol Chem Sci. 2022;13(3):138–44.

3. Fois AG, Paliogiannis P, Scano V, Cau S, Babudieri S, Perra R, et al. The Systemic Inflammation Index on Admission Predicts In-Hospital Mortality in COVID-19 Patients. Molecules. 2020 Dec 4;25(23):5725.

4. Younus S, Maqsood H, Sattar A, Younas A, Shakeel HA. A novel chest CT severity score in COVID-19 and its correlation with severity and prognosis of the lung disease: A retrospective cohort study. Ann Med Surg (Lond). 2022 Sep 15;82:104692.

5. Vishwanath T.T., Rajalakshmi B.R., Sadananda K.S., Manjunath C.N. Association of Chest CT Severity Scores and Vaccination Status in COVID-19 Disease: A Cross-sectional Study. J Clin Diagn Res. 2022;16(2):TC17–20.

6. Lin Q, Wu L, Lin W, Zhu L, Hu Y, Lin G, et al. Correlation between chest CT severity score and laboratory indicators in patients with Coronavirus disease 2019 (COVID-19). Int J Clin Pract. 2021 Dec;75(12):e14907.

7. De Smet D., De Smet K., Herroelen P., Gryspeerdt S., Martens G.A. Serum 25(OH)D Level on Hospital Admission Associated With COVID-19 Stage and Mortality. Am J Clin Pathol. 2020;((De Smet, Herroelen, Martens) AZ Delta Medical Laboratories, Roeselare, Belgium).

8. Yazdi NA, Ghadery AH, Seyedalinaghi SA, Jafari F, Jafari S, Hasannezad M, et al. Predictors of the chest CT score in COVID-19 patients: a cross-sectional study. Virol J. 2021 Nov 18;18:225.

9. Arcari L, Ciolina F, Cacciotti L, Danti M, Camastra G, Manzo D, et al. Semiquantitative Chest CT Severity Score Predicts Failure of Noninvasive Positive-Pressure Ventilation in Patients Hospitalized for COVID-19 Pneumonia. J Cardiothorac Vasc Anesth. 2022 Aug;36(8):2278–86.

10. Inamdar A., Saboo B. IDF21-0411 Correlation of Chest CT severity score with blood glucose levels in patients with COVID-19. Diabetes Res Clin Pract. 2022;186(Supplement 1):109351.

11. Zhou S, Chen C, Hu Y, Lv W, Ai T, Xia L. Chest CT imaging features and severity scores as biomarkers for prognostic prediction in patients with COVID-19. Ann Transl Med. 2020 Nov;8(21):1449.

12. Valk C., Zimatore C., Mazzinari G., Pierrakos C., Sivakorn C., Dechsanga J., et al. The RALE-score versus the CT Severity Score in Invasively Ventilated COVID-19 patients-a retrospective study comparing their prognostic capacities. Intensive Care Med Exp. 2022;10(Supplement 29).

13. Abbasi B, Akhavan R, Ghamari Khameneh A, Zandi B, Farrokh D, Pezeshki Rad M, et al. Evaluation of the relationship between inpatient COVID-19 mortality and chest CT severity score. Am J Emerg Med. 2021;45:458–63.

14. Halmaciu I, Arbănași EM, Kaller R, Mureșan AV, Arbănași EM, Bacalbasa N, et al. Chest CT Severity Score and Systemic Inflammatory Biomarkers as Predictors of the Need for Invasive Mechanical Ventilation and of COVID-19 Patients' Mortality. Diagnostics (Basel). 2022 Aug 29;12(9):2089.

15. Abd El Megid AGI, El Shabrawy M, Abdalla AAEHM. Correlation between chest CT severity scoring system with oxygen saturation and laboratory inflammatory markers in adult patients with COVID-19 infection. Egyptian Journal of Radiology and Nuclear Medicine. 2022 Mar 17;53(1):72.

16. Atre A.L., Atre A., Panchawagh S., Khamkar R., Chandorkar A., Patil S. Association between Chest CT Severity Scores and SARS-CoV-2 Vaccination among COVID-19 patients: A Cross-sectional Study from Pune, India. J Clin Diagn Res. 2022;16(6):TC11–7.

17. Aziz-Ahari A., Keyhanian M., Mamishi S., Mahmoudi S., Bastani E.E., Asadi F., et al. Chest CT severity score: assessment of COVID-19 severity and short-term prognosis in hospitalized Iranian patients. Wien Med Wochenschr. 2022;172(3–4):77–83.

18. Saeed GA, Gaba W, Shah A, Al Helali AA, Raidullah E, Al Ali AB, et al. Correlation between Chest CT Severity Scores and the Clinical Parameters of Adult Patients with COVID-19 Pneumonia. Radiology Research and Practice. 2021 Jan 8;2021:e6697677.

19. Padelli M, Gueye P, Guilloux D, Banydeen R, Campana V, Cabie A, et al. Soluble urokinase plasminogen activator receptor levels are predictive of COVID-19 severity in Afro-Caribbean patients. Biomark Med. :10.2217/bmm-2021–0669.

20. Sharma S, Aggarwal A, Sharma RK, Patras E, Singhal A. Correlation of chest CT severity score with clinical parameters in COVID-19 pulmonary disease in a tertiary care hospital in Delhi during the pandemic period. The Egyptian Journal of Radiology and Nuclear Medicine. 2022;53(1):166.
